# Protein Kinases Mediate Anti-Inflammatory Effects of Cannabidiol and Estradiol Against High Glucose in Cardiac Sodium Channels

**DOI:** 10.3389/fphar.2021.668657

**Published:** 2021-04-28

**Authors:** Mohamed A. Fouda, Peter C. Ruben

**Affiliations:** ^1^ Department of Biomedical Physiology and Kinesiology, Simon Fraser University, Burnaby, BC, Canada; ^2^ Department of Pharmacology and Toxicology, Alexandria University, Alexandria, Egypt

**Keywords:** diabetes, high glucose, sodium ion channels, inflammation, cannabidiol, estradiol, protein kinase A, protein kinase C

## Abstract

**Background:** Cardiovascular anomalies are predisposing factors for diabetes-induced morbidity and mortality. Recently, we showed that high glucose induces changes in the biophysical properties of the cardiac voltage-gated sodium channel (Nav1.5) that could be strongly correlated to diabetes-induced arrhythmia. However, the mechanisms underlying hyperglycemia-induced inflammation, and how inflammation provokes cardiac arrhythmia, are not well understood. We hypothesized that inflammation could mediate the high glucose-induced biophyscial changes on Nav1.5 through protein phosphorylation by protein kinases A and C. We also hypothesized that this signaling pathway is, at least partly, involved in the cardiprotective effects of cannabidiol (CBD) and 17β-estradiol (E_2_).

**Methods and Results:** To test these ideas, we used Chinese hamster ovarian (CHO) cells transiently co-transfected with cDNA encoding human Nav1.5 α-subunit under control, a cocktail of inflammatory mediators or 100 mM glucose conditions (for 24 h). We used electrophysiological experiments and action potential modeling. Inflammatory mediators, similar to 100 mM glucose, right shifted the voltage dependence of conductance and steady-state fast inactivation and increased persistent current leading to computational prolongation of action potential (hyperexcitability) which could result in long QT3 arrhythmia. We also used human iCell cardiomyocytes derived from inducible pluripotent stem cells (iPSC-CMs) as a physiologically relevant system, and they replicated the effects produced by inflammatory mediators observed in CHO cells. In addition, activators of PK-A or PK-C replicated the inflammation-induced gating changes of Nav1.5. Inhibitors of PK-A or PK-C, CBD or E_2_ mitigated all the potentially deleterious effects provoked by high glucose/inflammation.

**Conclusion:** These findings suggest that PK-A and PK-C may mediate the anti-inflammatory effects of CBD and E_2_ against high glucose-induced arrhythmia. CBD, via Nav1.5, may be a cardioprotective therapeutic approach in diabetic postmenopausal population.

## Introduction

Cardiovascular anomalies are strongly correlated with diabetes-induced morbidity and mortality (Matheus et al., 2013). These deleterious cardiovascular complications are mainly attributed to hyperglycemia/high glucose (Pistrosch et al., 2011). There is also a positive correlation between diabetes/high glucose and long QT (LQT) syndrome (Grisanti, 2018; Fouda et al., 2020a). LQT syndrome is a cardiac arrhythmogenic disorder, identified by a prolongation of the Q-T interval. One cause of LQT syndrome is a gain-of-function in cardiac sodium channels, as in LQT3 (Shimizu and Antzelevitch, 1999).

Oxidative stress and activation of pro-inflammatory pathways are among the main pathways involved in diabetes/high glucose evoked cardiovascular abnormalities (Rajesh et al., 2010). Cardiac inflammation has a key role in the development of cardiovascular anomalies (Adamo et al., 2020). Inhibition of inflammatory signaling pathways ameliorate cardiac consequences (Adamo et al., 2020). Ion channels are crucial players in inflammation-induced cardiac abnormalities (Eisenhut and Wallace, 2011). Voltage-gated sodium channels (Nav) underlie phase 0 of the cardiac action potential (Balser, 1999; Ruan et al., 2009). Changes in the biophysical properties of the primary cardiac sodium channel, Nav1.5, are linked to diabetes induced cardiovascular abnormalities (Yu et al., 2018; Fouda et al., 2020a). However, the mechanisms underlying hyperglycemia-induced inflammation, and how inflammation provokes cardiac dysfunction, are not well understood.

Cannabidiol (CBD) is approved as an anti-seizure drug (Barnes, 2006; Devinsky et al., 2017). CBD lacks adverse cardiac toxicity and ameliorates diabetes/high glucose induced deletrious cardiomyopathy (Cunha et al., 1980; Izzo et al., 2009; Rajesh et al., 2010). Recently, we showed that CBD rescues the biophysical substrate for LQT3 via direct inhibitory effects on cardiac sodium ion channels and indirect anti-oxidant effects (Fouda et al., 2020a). In addition, CBD inhibits the production of pro-inflammatory cytokines *in vitro* and *in vivo* (Nichols and Kaplan, 2020).

Gonadal hormones have crucial roles in the inflammatory responses (El-Lakany et al., 2018; El-Lakany et al., 2020). Estrogen (E_2_), the main female sex hormone, acts via genomic and non-genomic mechanisms to inhibit inflammatory cascades (Murphy et al., 2010). Clinically, postmenopausal females exhibited higher levels of TNF-α in reponse to endotoxemia compared with pre-menopausal women (Moxley et al., 2004). Interestingly, E_2_ stabilizes Nav fast inactivation and reduces the late sodium currents (Wang et al., 2010), similar to CBD effects on Nav1.5 (Fouda et al., 2020a).

Here, we characterized the role of inflammation in high glucose-induced biophyscial changes on Nav1.5. Second, we found that changes in the biophysical properties of Nav1.5 may be, at least in part, mediated through protein phosphorylation by protein kinases A and C. Finally, we show that this signaling pathway may be, at least partly, involved in the cardiprotective effects of CBD and E_2_.

## Materials and Methods

### Cell Culture

Chinese hamster ovary cells (CHO) (RRID: CVCL_0214; passages from 3–13) were grown at pH 7.4 in filtered sterile F12 (Ham’s) nutrient medium (Life Technologies, Thermo Fisher Scientific, Waltham, MA, United States), supplemented with 5% FBS and maintained in a humidified environment at 37°C with 5% CO_2_. Cells were transiently co-transfected with the human cDNA encoding the Nav1.5 α-subunit, the β1-subunit, and eGFP. Transfection was done according to the PolyFect (Qiagen, Germantown, MD, United States) transfection protocol. A minimum of 8°h incubation was allowed after each set of transfections. The cells were subsequently dissociated with 0.25% trypsin–EDTA (Life Technologies, Thermo Fisher Scientific) and plated on sterile coverslips under normal (10 mM) or elevated glucose concentrations (100 mM) (Fouda et al., 2020a) or a cocktail of inflammatory mediators (Akin et al., 2019) containing bradykinin (1 µM), PGE-2 (10 µM), histamine (10 µM), 5-HT (10 µM), and adenosine 5′-triphosphate (15 µM) for 24 h prior to electrophysiological experiments. We used 10 mM (control, and the necessary concentration for cultured CHO cell viability) and 100 mM glucose to ensure sufficiently large readout signals throughout the study; we recognize these are not physiologically relevant concentrations. It should be noted that hyperglycemia is the most important factor in the onset and progress of diabetic complications (Viskupicova et al., 2015). High glucose concentrations are usually used as a model to mimic the *in vivo* situation of hyperglycemia in diabetes (Viskupicova et al., 2015). High glucose concentrations (up to 100 mM of D-glucose) have been used previously to mimic the human hyperglycemia based on the used cell line (Viskupicova et al., 2015). 100 mM glucose was used in many studies and in different cell lines, including human erythrocytes (incubation for 72 h) (Viskupicova et al., 2015), human SH-SY5Y neuroblastoma cell line (Liu et al., 2019), and neuronal PC 12 cells (Chen et al., 2016; Fouda and Abdel-Rahman, 2017). We have previously used the MTS cell viability assay to optimize the glucose concentration that would mimic the diabetic/hyperglycemia conditions in CHO cells (Fouda et al., 2020a).

Importantly, it should be noted that CHO cells endogenously express the receptors for the abovementioned inflammatory mediators (Fukushima et al., 1994; George et al., 1997; Zhang et al., 2001; de Wilde et al., 2010).

### iCell Cardiomyocytes

Single vials containing ≥1 × 10^6^ iCell cardiomyocytes (Fujifilm Cellular Dynamics International, kit R1105, Madison, WI, United States ) were thawed by immersing the frozen cryovial in a 37°C water bath, transferring thawed iCell cardiomyocytes into a 50°ml tube, and diluting them with 10°ml of ice-cold plating medium (iCell Cardiomyocytes Plating Medium (iCPM); Fujifilm Cellular Dynamics International, Madison, WI, United States) (Ma et al., 2011). For single cell patch-clamp recordings, glass coverslips were coated with 0.1% gelatin (Fujifilm Cellular Dynamics International, Madison, WI, United States) and placed into each well of a 24-well plate for an hour. This was followed by adding 1°ml of iCPM containing 40,000–60,000 iCell cardiomyocytes to each coverslip. Plated iCell cardiomyocytes were at a low density to permit culture as single cells and were stored in an environmentally controlled incubator maintained at 37°C and 7% CO_2_. After 48°h, iCPM was replaced with a cell culture medium (iCell Cardiomyocytes Maintenance Medium (iCMM); Fujifilm Cellular Dynamics International, Madison, WI, United States), which was exchanged every other day with the iCell cardiomyocytes maintained on cover slips for 4–21°days before use (Ma et al., 2011). iCell cardiomyocytes were incubated in a cocktail of inflammatory mediators (Akin et al., 2019) containing bradykinin (1 µM), PGE-2 (10 µM), histamine (10 µM), 5-HT (10 µM), and adenosine 5′-triphosphate (15 µM) or the vehicle for 24 h prior to electrophysiological experiments.

### Electrophysiology

Whole-cell patch clamp recordings from Nav1.5 expressed in CHO cells were made using an extracellular solution composed of NaCl (140 mM), KCl (4 mM), CaCl_2_ (2 mM), MgCl_2_ (1 mM), HEPES (10 mM). The extracellular solution was titrated to pH 7.4 with CsOH. Pipettes were fabricated with a P-1000 puller using borosilicate glass (Sutter Instruments, CA, United States), dipped in dental wax to reduce capacitance, then thermally polished to a resistance of 1.0–1.5 MΩ. Pipettes were filled with intracellular solution, containing: CsF (120 mM), CsCl (20 mM), NaCl (10 mM), HEPES (10 mM) titrated to pH 7.4. Patch clamp recordings on human iCell cardiomyocytes were made using an extracellular solution composed of NaCl (50 mM), CaCl_2_ (1.8 mM), MgCl_2_ (1 mM), CsCl_2_ (110 mM), glucose (10 mM), HEPES (10 mM) and nifedipine (0.001 mM) (Ma et al., 2011). The extracellular solution was titrated to pH 7.4 with CsOH. Pipettes were fabricated with a P-1000 puller using borosilicate glass (Sutter Instruments, CA, United States), dipped in dental wax to reduce capacitance, then thermally polished to a resistance of 2.0–3.5 MΩ. Pipettes were filled with intracellular solution, containing: CsCl_2_ (135 mM), NaCl (10 mM), CaCl_2_ (2 mM), EGTA (5 mM), HEPES (10 mM) and Mg-ATP (5 mM) titrated to pH 7.2 with CsOH (Ma et al., 2011).

All recordings were made using an EPC-9 patch-clamp amplifier (HEKA Elektronik, Lambrecht, Germany) digitized at 20 kHz via an ITC-16 interface (Instrutech, Great Neck, NY, United States). Voltage clamping and data acquisition were controlled using PatchMaster/FitMaster software (HEKA Elektronik, Lambrecht, Germany) running on an Apple iMac (Cupertino, California). Current was low-pass-filtered at 5 kHz. Leak subtraction was automatically done using a p/4 procedure following the test pulse. Gigaohm seals were allowed to stabilize in the on-cell configuration for 1 min prior to establishing the whole-cell configuration. Series resistance was less than 5 MΩ for all recordings. Series resistance compensation up to 80% was used when necessary. All data were acquired at least 5 min after attaining the whole-cell configuration, and cells were allowed to incubate 5 min after drug application prior to data collection. Before each protocol, the membrane potential was hyperpolarized to −130 mV for 197 ms to insure complete removal of both fast-inactivation and slow-inactivation. Leakage and capacitive currents were subtracted with a P/4 protocol. All experiments were conducted at 22°C.

### Activation Protocols

To determine the voltage-dependence of activation, we measured the peak current amplitude at test pulse voltages ranging from −130 to + 80 mV in increments of 10 mV for 19 ms. Channel conductance (G) was calculated from peak I_Na_:

$$G_{\mathrm{Na}}=I_{\mathrm{Na}}/\left(V-E_{\mathrm{Na}}\right)$$ (1)

where $G_{\mathrm{Na}}$ is conductance, $I_{\mathrm{Na}}$ is peak sodium current in response to the command potential V, and $E_{\mathrm{Na}}$ is the Nernst equilibrium potential. The midpoint and apparent valence of activation were derived by plotting normalized conductance as a function of test potential. Data were then fitted with a Boltzmann function:

$$G/G_{\max}=1/\left\{1+\exp\left[-{\mathrm{ze}}_{0}\left(V_{m}-V_{1/2}\right)/\mathrm{KT}\right]\right\}$$ (2)

where $G/G_{\max}$ is normalized conductance amplitude, Vm is the command potential, z is the apparent valence, e_0_ is the elementary charge, $V_{1/2}$ is the midpoint voltage, k is the Boltzmann constant, and T is temperature in K.

### Steady-State Fast Inactivation Protocols

The voltage-dependence of fast-inactivation was measured by preconditioning the channels to a hyperpolarizing potential of −130 mV (to insure complete channel availability) and then eliciting pre-pulse potentials that ranged from −170 to + 10 mV in increments of 10 mV for 500 ms, followed by a 10 ms test pulse during which the voltage was stepped to 0 mV. Normalized current amplitude as a function of voltage was fit using the Boltzmann function:

$$I/I_{\max}=1/\left\{1+\exp\left[-{\mathrm{ze}}_{0}\left(V_{m}-V_{1/2}\right)/\mathrm{KT}\right]\right\}$$ (3)

where I_max_ is the maximum test pulse current amplitude. z is apparent valence, e_0_ is the elementary charge, Vm is the prepulse potential, $V_{1/2}$ is the midpoint voltage of SSFI, k is the Boltzmann constant, and T is temperature in K.

### Fast Inactivation Recovery

Channels were fast inactivated during a 500 ms depolarizing step to 0 mV. Recovery was measured during a 19 ms test pulse to 0 mV following −130 mV (to insure complete channel availability) recovery pulse for durations between 0 and 1.024 s. Time constants of fast inactivation were derived using a double exponential equation:

$$\mathrm{I=}I_{\mathrm{ss}}+\alpha_{1}\exp\left(-t/\tau_{1}\right)+\alpha_{2}\exp\left(-t/\tau_{2}\right)$$ (4)

where I is current amplitude, $I_{\mathrm{ss}}$ is the plateau amplitude, α1 and α2 are the amplitudes at time 0 for time constants τ_1_ and τ_2_, and t is time.

### Persistent Current Protocols

Late sodium current was measured between 45 and 50 ms during a 50 ms or between 145 and 150 ms during a 200 ms depolarizing pulse to 0 mV from a holding potential of −130 mV (to insure complete channel availability) on CHO cells or iCell cardiomyocytes, respectively. Fifty pulses were averaged to increase signal to noise ratio (Abdelsayed et al., 2015; Abdelsayed et al., 2018).

### Action Potential Modeling

Action potentials were simulated using a modified version of the O’Hara-Rudy model programmmed in Matlab (O’Hara et al., 2011, PLoS Comput. Bio). The code that was used to produce model is available online from the Rudy Lab website (http://rudylab.wustl.edu/research/cell/code/Allcodes.html). The modified gating INa parameters were in accordance with the biophysical data obtained from whole-cell patch-clamp experiments in this study for various conditions. The model accounted for activation voltage-dependence, steady-state fast inactivation voltage-dependence, persistent sodium currents, and peak sodium currents (compound conditions).

### Drug Preparations

CBD was purchased from Toronto Research Chemicals (Toronto, Ontario) in powder form. Other compounds (e.g., 17β-estradiol (E_2_), bradykinin, PGE-2, histamine, 5-HT, adenosine 5′-triphosphate, D-glucose, Gö 6983 (PKC inhibitor), H-89 (PKA inhibitor), 8-(4-chlorophenylthio) adenosine- 3′,5′-cyclic monophosphate (CPT-cAMP; PKA activator) or PMA (PKC activator)) were purchased from Sigma-Aldrich (ON, Canada). Powdered CBD, Gö 6983, H-89, adenosine CPT-cAMP or PMA were dissolved in 100% DMSO to create stock and kept frozen until use. The stock was used to prepare drug solutions in extracellular solutions at various concentrations, immediately prior to perfusion, with no more than 0.5% total DMSO content [which has no effect on sodium currents (Ghovanloo et al., 2018; Fouda et al., 2020a)]. Stock solution of E_2_ (1 mM) was prepared in phosphate buffered saline (PBS) to be freshely diluted to 5 or 10 µM using the external solution in the day of the electrophysiological experiment.

### Data Analysis and Statistics

Studies were designed to generate groups of almost equal size (*n* = 5), using randomization and blinded analysis. Normalization was performed in order to control the variations in sodium channel expression and inward current amplitude and in order to be able to fit the recorded data with Boltzmann function (for voltage-dependences) or an exponential function (for time courses of inactivation). Fitting and graphing were done using FitMaster software (HEKA Elektronik, Lambrecht, Germany) and Igor Pro (Wavemetrics, Lake Oswego, OR, United States). Statistical analysis consisted of one-way ANOVA (endpoint data) along with post hoc testing of significant findings along with Student’s t-test and Tukey’s test using Prism 7 software (Graphpad Software Inc., San Diego, CA, United States). Values are presented as mean ± SEM with probability levels less than 0.05 considered significant. Statistical analysis was undertaken only for studies where each group size was at least “*n* = 5.” The declared group size is the number of independent values, and that statistical analysis was done using these independent values. In the electrophysiological experiments, we randomized the different treatments under the different conditions (e.g., control vs. high glucose or inflammatory mediators), so that five cells in each treatment or condition came from five different randomized cell passages.

## Results

### Inflammatory Mediators Alter the Gating Properties of Nav1.5 Similar to High Glucose

**FIGURE 1:**
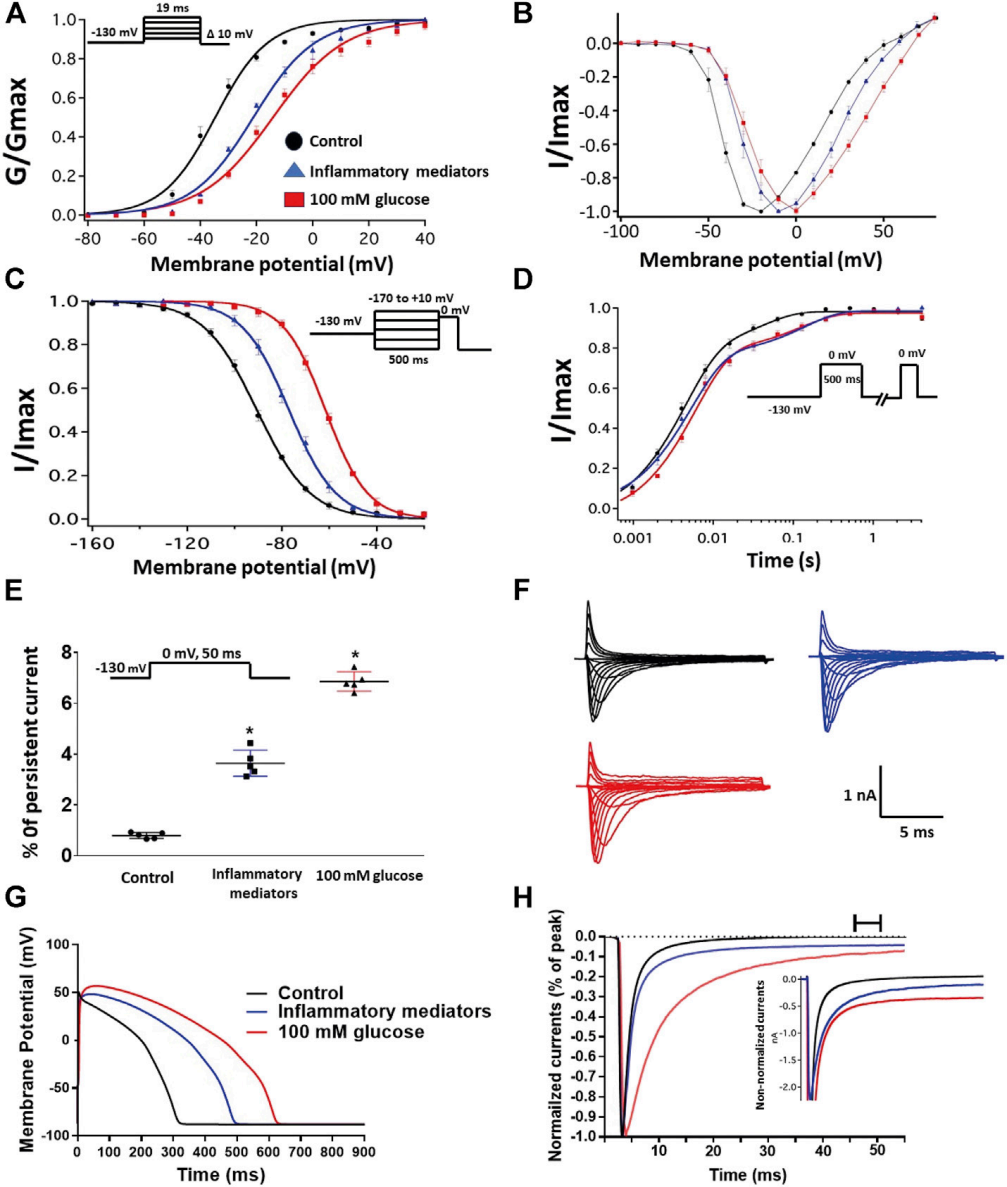
**(A)** Effect of a cocktail of inflammatory mediators or 100 mM glucose or their vehicle (for 24 h) on conductance curve of Nav1.5 transfected CHO cells with the insert showing the protocol (*n* = 5, each). **(B)** IV curves **(C)** Effect of a cocktail of inflammatory mediators or 100 mM glucose or their vehicle (for 24 h) on SSFI of Nav1.5 transfected CHO cells with the insert showing the protocol (*n* = 5, each). **(D)** Effect of a cocktail of inflammatory mediators or 100 mM glucose or their vehicle (for 24 h) on recovery from fast inactivation of Nav1.5 transfected CHO cells with the insert showing the protocol (*n* = 5, each). **(E)** Effect of a cocktail of inflammatory mediators or 100 mM glucose or their vehicle (for 24 h) on the percentage of persistent sodium currents of Nav1.5 transfected CHO cells with the insert showing the protocol (*n* = 5, each). **(F)** Representative families of macroscopic currents. **(G)** *In silico* action potential duration of Nav1.5 transfected CHO cells incubated in inflammatory mediators or 100 mM glucose or the vehicle for 24 h. **(H)** Representative persistent currents across conditions. Currents were normalized to peak current amplitude. Bar above current traces indicates period during which persistent current was measured. Inset shows non-normalized currents. *p < 0.05 vs. corresponding “Control” values using one-way ANOVA along with post hoc testing.

We recently showed that high glucose, in a concentration-dependent manner, right-shifts the voltage dependence of activation and steady-state fast inactivation and increases persistent current (Fouda et al., 2020a). Here, we used whole-cell voltage-clamp to measure gating in human Nav1.5, and test the effects of incubating for 24 h in either a cocktail of inflammatory mediators (Akin et al., 2019) or 100 mM glucose (Fouda et al., 2020a). Peak channel conductance was measured between −130 and + 80 mV. We measured channel conductance in the presence of inflammatory mediators to determine whether the high glucose induced-changes in Nav1.5 activation (Fouda et al., 2020a) are, at least partly, mediated through inflammation. Figures 1A,B show the conductance plotted as a function of membrane potential and the current-voltage (IV) curve, respectively. High glucose (100 mM) significantly shifted the Nav1.5 midpoint (V_1/2_) of activation in the positive direction (*p* = 0.0002). Additionally, the slope (apparent valence, z) of the activation curves showed a significant decrease in 100 mM glucose (*p* = 0.007) (Figure 1A; Table 1). This decrease in slope suggests a reduction in activation charge sensitivity. We found that incubation in inflammatory mediators for 24 h, similar to 100 mM glucose, significantly right-shifted V_1/2_ of activation (*p* = 0.001) and decreased z of activation curve (*p* = 0.03) (Figure 1A; Table 1). This suggests that both 100 mM glucose or inflammatory mediators decrease the probability of Nav1.5 activation.

**TABLE 1:** Steady-state activation.

	GV−V_1/2_ (mV)	GV−z (slope)	Current density (pA/pF)	*n*
			(at 0 mV)	
Control				
Control/Vehicle	−36.2 ± 1.6	3.3 ± 0.2	−836.2 ± 83.1	5
Vehicle/H-89	−39.6 ± 1.4	3.2 ± 0.1	−832.5 ± 77.2	5
Vehicle/Gö 6983	−37.7 ± 0.7	3.1 ± 0.1	−877.3 ± 78.8	5
Glucose (100 mM)				
100 mM glucose/Vehicle	−16.6 ± 2.8	2.5 ± 0.1	−899.9 ± 115.7	5
Inflammatory mediators (IM)				
IM/Vehicle	−22.3 ± 2.4	2.7 ± 0.2	−761.8 ± 36.1	5
IM/H-89	−32.7 ± 1.4	2.8 ± 0.2	−661.8 ± 99.3	5
IM/Gö 6983	−31.7 ± 1.6	2.7 ± 0.1	−714.0 ± 116.9	5
CPT- cAMP	−25.2 ± 0.5	2.5 ± 0.1	−697.3 ± 120.0	5
PMA	−22.6 ± 1.6	2.5 ± 0.1	−797.2 ± 119.1	5
CBD (5 µM)				
IM/CBD	−39.1 ± 2.8	3.6 ± 0.1	−462.3 ± 32.7	5
CPT-cAMP/CBD	−33.1 ± 0.6	3.4 ± 0.1	−414.6 ± 56.2	5
PMA/CBD	−35.3 ± 0.9	3.3 ± 0.1	−437.5 ± 43.6	5
E2				
E_2_ 5°µM/vehicle	−34.8 ± 1.5	3.1 ± 0.1	−709.5 ± 30.3	5
E_2_ 10°µM/vehicle	−34.3 ± 0.9	3.0 ± 0.1	−729.5 ± 49.3	5
E_2_ 5°µM/glucose 100 mM	−27.3 ± 0.7	2.4 ± 0.1	−856.3 ± 21.7	5
E_2_ 10°µM/glucose 100 mM	−37.9 ± 1.4	3.5 ± 0.1	−893.4 ± 71.8	5
E_2_ 5°µM/IM	−29.8 ± 1.3	2.8 ± 0.1	−730.2 ± 39.5	5
E_2_ 10°µM/IM	−35.7 ± 2.0	3.5 ± 0.1	−788.7 ± 28.5	5
E_2_ 10°µM/CPT-cAMP	−37.7 ± 0.6	3.6 ± 0.1	−760.2 ± 74.4	5
E_2_ 10°µM/PMA	−35.9 ± 1.5	3.4 ± 0.2	−808.5 ± 55.0	5

The DIII-IV linker mediates fast inactivation within a few milliseconds of Nav activation (West et al., 1992). Figure 1C shows normalized current amplitudes plotted as a function of pre-pulse potential. 100 mM glucose or inflammatory mediators caused significant shifts in the positive direction in the V_1/2_ obtained from Boltzmann fits (100 mM glucose: *p* < 0.0001; inflammatory mediators: *p* = 0.001) (Figure 1C; Table 2). These shifts indicate a loss-of-function in fast inactivation and suggest that high glucose or inflammatory mediators decrease the probability of steady-state fast inactivation (SSFI) in Nav1.5.

**TABLE 2:** Steady-state fast inactivation.

	SSFI−V_1/2_ (mV)	SSFI−z (slope)	*n*
Control			
Control/Vehicle	−90.9 ± 1.8	−2.6 ± 0.1	5
Vehicle/H-89	−89.3 ± 1.9	−2.7 ± 0.1	5
Vehicle/Gö 6983	−88.6 ± 2.1	−3.0 ± 0.1	5
Glucose (100 mM)			
100 mM glucose/Vehicle	−61.7 ± 2.6	−2.9 ± 0.1	5
Inflammatory mediators (IM)			
IM/Vehicle	−77.1 ± 1.7	−2.6 ± 0.1	5
IM/H-89	−86.4 ± 2.8	−2.9 ± 0.2	5
IM/Gö 6983	−87.1 ± 2.0	−2.4 ± 0.2	5
CPT-cAMP	−79.4 ± 1.1	−3.0 ± 0.1	5
PMA	−76.4 ± 1.7	−2.9 ± 0.2	5
CBD (5 µM)			
IM/CBD	−85.9 ± 1.5	−2.6 ± 0.3	5
CPT-cAMP/CBD	−86.8 ± 2.3	−2.9 ± 0.2	5
PMA/CBD	−85.7 ± 1.2	−2.9 ± 0.1	5
E2			
E_2_ 5°µM/vehicle	−87.4 ± 2.1	−2.8 ± 0.1	5
E_2_ 10°µM/vehicle	−87.6 ± 2.1	−3.0 ± 0.2	5
E_2_ 5°µM/glucose 100 mM	−75.5 ± 1.9	−2.8 ± 0.2	5
E_2_ 10°µM/glucose 100 mM	−91.1 ± 3.6	−2.8 ± 0.1	5
E_2_ 5°µM/IM	−81.1 ± 2.1	−2.8 ± 0.1	5
E_2_ 10°µM/IM	−92.6 ± 0.8	−2.6 ± 0.1	5
E_2_ 10°µM/CPT-cAMP	−89.3 ± 1.9	−2.6 ± 0.1	5
E_2_ 10°µM/PMA	−88.7 ± 0.6	−2.3 ± 0.2	5

To measure fast inactivation recovery, we held channels at −130 mV to ensure channels were fully at rest, then depolarized the channels to 0 mV for 500 ms, and allowed different time intervals at −130 mV to measure recovery as a function of time. We found that incubation in 100 mM glucose or inflammatory mediators significantly (*p* < 0.05) increase the slow component of fast inactivation recovery when compared to control, without affecting the fast component of recovery (Figure 1D; Table 3).

**TABLE 3:** Time constants for the recovery from fast inactivation.

	τ fast (s)	τ slow (s)	*n*
Control			
Control/Vehicle	0.006 ± 0.001	0.006 ± 0.001	5
Vehicle/H-89	0.007 ± 0.001	0.006 ± 0.001	5
Vehicle/Gö 6983	0.006 ± 0.001	0.010 ± 0.002	
Glucose (100 mM)			
100 mM glucose/Vehicle	0.008 ± 0.002	0.111 ± 0.03	5
Inflammatory mediators (IM)			
IM/Vehicle	0.005 ± 0.001	0.123 ± 0.002	5
IM/H-89	0.010 ± 0.002	0.303 ± 0.036	5
IM/Gö 6983	0.008 ± 0.002	0.304 ± 0.031	5
CPT- cAMP	0.006 ± 0.001	0.168 ± 0.009	5
PMA	0.005 ± 0.001	0.175 ± 0.005	5
CBD (5 µM)			
IM/CBD	0.008 ± 0.001	0.209 ± 0.020	5
CPT-cAMP/CBD	0.009 ± 0.001	0.207 ± 0.004	5
PMA/CBD	0.006 ± 0.001	0.218 ± 0.014	5
E_2_			
E_2_ 5°µM/vehicle	0.006 ± 0.001	0.011 ± 0.002	5
E_2_ 10°µM/vehicle	0.006 ± 0.001	0.010 ± 0.002	5
E_2_ 5°µM/glucose 100 mM	0.005 ± 0.001	0.148 ± 0.009	5
E_2_ 10°µM/glucose 100 mM	0.008 ± 0.002	0.228 ± 0.015	5
E_2_ 5°µM/IM	0.005 ± 0.001	0.182 ± 0.015	5
E_2_ 10°µM/IM	0.005 ± 0.001	0.262 ± 0.015	5
E_2_ 10°µM/CPT-cAMP	0.007 ± 0.001	0.222 ± 0.008	5
E_2_ 10°µM/PMA	0.007 ± 0.001	0.233 ± 0.006	5

An increased persistent sodium current (INap) is a manifestation of destabilized fast inactivation (Goldin, 2003). Large INap is associated with a range of pathological conditions, including LQT3 (Wang et al., 1995; Ghovanloo et al., 2016). To determine the effects of glucose or inflammatory mediators on the stability of Nav1.5 inactivation, we held channels at −130 mV, followed by a depolarizing pulse to 0 mV for 50 ms (Abdelsayed et al., 2015; Abdelsayed et al., 2018). Figure 1E shows that incubation in 100 mM glucose or inflammatory mediators significantly increased INap compared to control (100 mM glucose: *p* < 0.0001; inflammatory mediators: *p* < 0.0001) (Table 4). Representative families of macroscopic and persistent currents across conditions are shown (Figures 1F,H). Notably, incubation in either high glucose (100 mM) or inflammatory mediators for 24 h had no significant effect on the current density of Nav1.5 when compared to the control condition (Table 1).

**TABLE 4:** Persistent current.

	Percentage of persistent I_Na_	*n*
Control		
Control/Vehicle	0.80 ± 0.05	5
Vehicle/H-89	0.82 ± 0.07	5
Vehicle/Gö 6983	0.84 ± 0.08	5
Glucose (100 mM)		
100 mM glucose/Vehicle	6.86 ± 0.17	5
Inflammatory mediators (IM)		
IM/Vehicle	3.64 ± 0.23	5
IM/H-89	1.21 ± 0.07	5
IM/Gö 6983	1.22 ± 0.06	5
CPT- cAMP	2.20 ± 0.08	5
PMA	2.18 ± 0.06	5
CBD (5 µM)		
IM/CBD	0.93 ± 0.05	5
CPT-cAMP/CBD	1.04 ± 0.11	5
PMA/CBD	0.88 ± 0.07	5
E2		
E2 5°µM/vehicle	0.85 ± 0.06	5
E2 10°µM/vehicle	0.91 ± 0.06	5
E2 5°µM/glucose 100 mM	1.92 ± 0.09	5
E2 10°µM/glucose 100 mM	0.89 ± 0.06	5
E_2_ 5°µM/IM	1.73 ± 0.03	5
E_2_ 10°µM/IM	0.85 ± 0.06	5
E_2_ 10°µM/CPT-cAMP	0.95 ± 0.09	5
E_2_ 10°µM/PMA	0.90 ± 0.11	5

We used the O’Hara-Rudy model to simulate cardiac action potentials (AP) (O’Hara et al., 2011). The sodium channel parameters in the model were modified using the results of our experiments and the effects of the tested compounds on the measured biophysical properties of activation (midpoint and apparent valence), steady-state fast inactivation (midpoint), recovery from fast inactivation, and persistent sodium current amplitude. The original model sodium channel parameters were adjusted to correspond to the control results from the patch-clamp experiments and the subsequent magnitude shifts in the simulations, caused by other conditions, were adjusted relative to the control parameters (Fouda et al., 2020a). Figure 1G shows that modifying the model with sodium channel parameters obtained from incubation in 100 mM glucose or inflammatory mediators prolonged the simulated AP duration (APD) from ∼300 to ∼500 ms (inflammatory mediators) and to >600 ms (100 mM glucose). This increased APD potentially leads to QT interval prolongation (Nachimuthu et al., 2012). Despite the similarity between 100 mM glucose and inflammatory mediators-induced changes on Nav1.5, their responses are not exactly the same (Figure 1). This could be attributed to the concentration-dependent effects of high glucose on electrophysiological properties of Nav1.5 (Fouda et al., 2020a).

### Activation of Protein Kinase A and Protein Kinase C Mediates the Inflammatory Mediators Induced Alteration in the Gating Properties of Nav1.5

**FIGURE 2:**
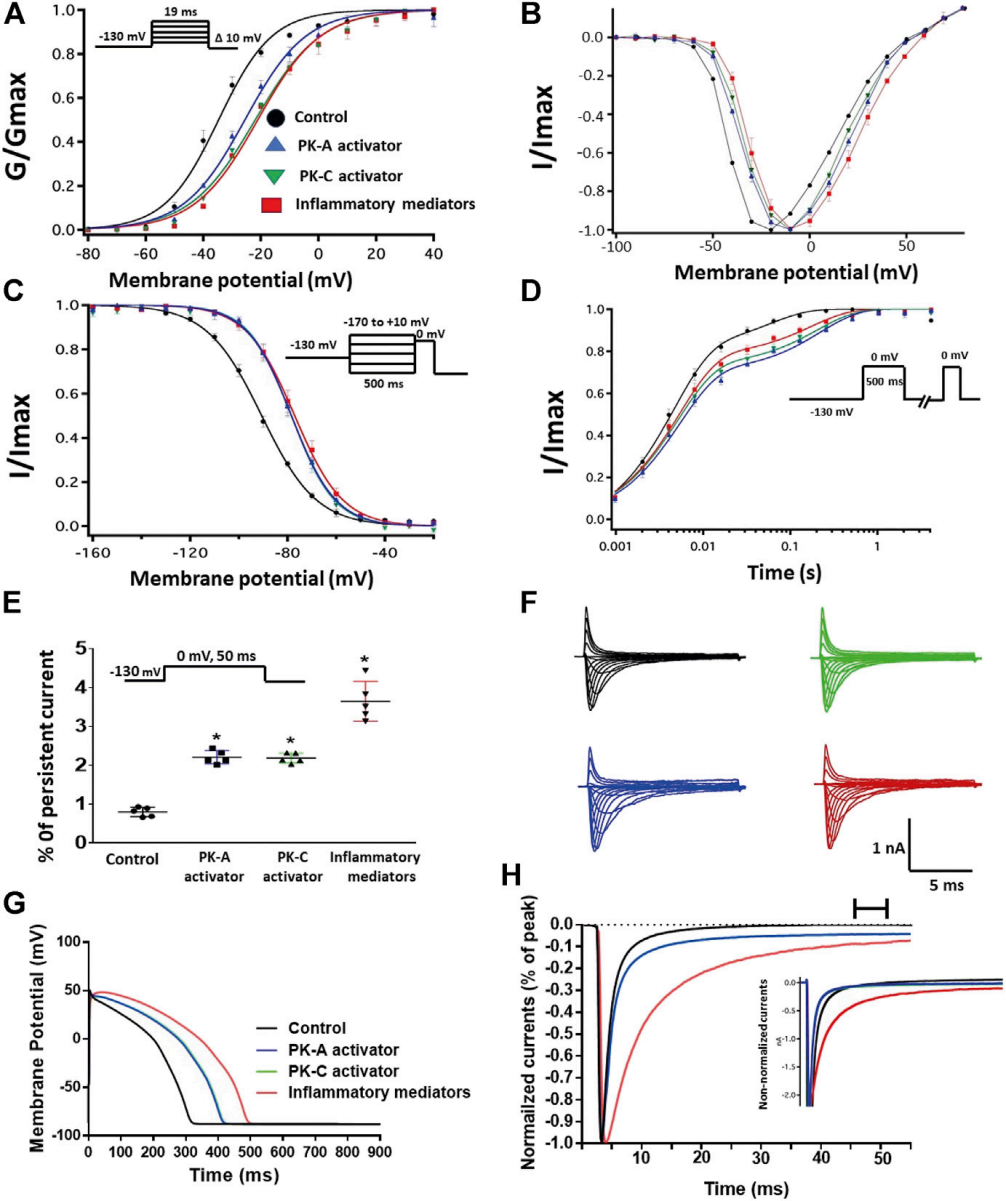
**(A)** Effect of inflammatory mediators (for 24 h) or PK-A activator (CPT-cAMP; 1 μM, for 20 min) or PK-C activator (PMA; 10 nM, for 20 min) on conductance curve Nav1.5 transfected CHO cells with the insert showing the protocol (n = 5, each). **(B)** IV curves **(C)** Effect of inflammatory mediators (for 24 h) or PK-A activator (CPT-cAMP; 1 μM, for 20 min) or PK-C activator (PMA; 10 nM, for 20 min) on SSFI of Nav1.5 transfected CHO cells with the insert showing the protocol (*n* = 5, each). **(D)** Effect of inflammatory mediators (for 24 h) or PK-A activator (CPT-cAMP; 1 μM, for 20 min) or PK-C activator (PMA; 10 nM, for 20 min) on recovery from fast inactivation of Nav1.5 transfected CHO cells with the insert showing the protocol (*n* = 5, each). **(E)** Effect of inflammatory mediators (for 24 h) or PK-A activator (CPT-cAMP; 1 μM, for 20 min) or PK-C activator (PMA; 10 nM, for 20 min) on the percentage of persistent sodium currents of Nav1.5 transfected CHO cells with the insert showing the protocol (*n* = 5, each). **(F)** Representative families of macroscopic currents. **(G)** Effect of PK-A activator (CPT-cAMP; 1 µM for 20 min), PK-C activator (PMA; 10 nM, for 20 min) or inflammatory mediators (for 24 h) on the *In silico* action potential duration of Nav1.5 transfected CHO cells. **(H)** Representative persistent currents across conditions. Currents were normalized to peak current amplitude. Bar above current traces indicates period during which persistent current was measured. Inset shows non-normalized currents. *p < 0.05 vs. corresponding “Control” values using one-way ANOVA along with post hoc testing.

One of the key signaling pathways involved in inflammation is the activation of protein kinase A or protein kinase C and subsequent protein phosphorylation (Karin, 2005). To pharmacologically investigate the role of PK-A or PK-C signaling pathways in the inflammation-evoked gating changes of Nav1.5, we recorded Nav1.5 currents at room temperature in the absence, or after a 20 min perfusion, of a PK-C activator [PMA; 10 nM (Hallaq et al., 2012)] or PK-A activator [CPT-cAMP; 1 µM (Ono et al., 1993; Gu et al., 2003)]. PMA or CPT-cAMP significantly shifted the Nav1.5 V_1/2_ of activation in the positive direction (PMA: *p* = 0.0003; CPT-cAMP: *p* = 0.0007) (Figure 2A; Table 1). In addition, PMA or CPT-cAMP significantly reduced the effective valence (z) of the activation curves (PMA: *p* = 0.002; CPT-cAMP: *p* = 0.007) (Figure 2A; Table 1). Figure 1B shows the IV curves. Furthermore, PMA or CPT-cAMP caused significant right-shifts in the V_1/2_ of SSFI (PMA: *p* = 0.0008; CPT-cAMP: *p* = 0.0005) (Figure 2C; Table 2). Also, PMA or CPT-cAMP significantly (*p* < 0.05) increase the slow component of fast inactivation recovery when compared to control (Figure 2D; Table 3). We also found that PMA or CPT-cAMP significantly (PMA: *p* < 0.0001; CPT-cAMP: *p* < 0.0001) increased INap compared to control (Figure 2E; Table 4). These effects are similar to those of glucose and inflammatory mediators (Figure 1). Representative families of macroscopic and persistent currents across conditions are shown (Figures 2F,H). Similar to 100 mM glucose and inflammatory mediators, the data from PK-A (CPT-cAMP) or PK-C (PMA) activator experiments shows that the *in silico* APD increased from ∼300 ms to ∼400 ms (Figure 2G). PMA or CPT-cAMP had no significant effect on the current density of Nav1.5 when compared to the control condition (Table 1).

**FIGURE 3:**
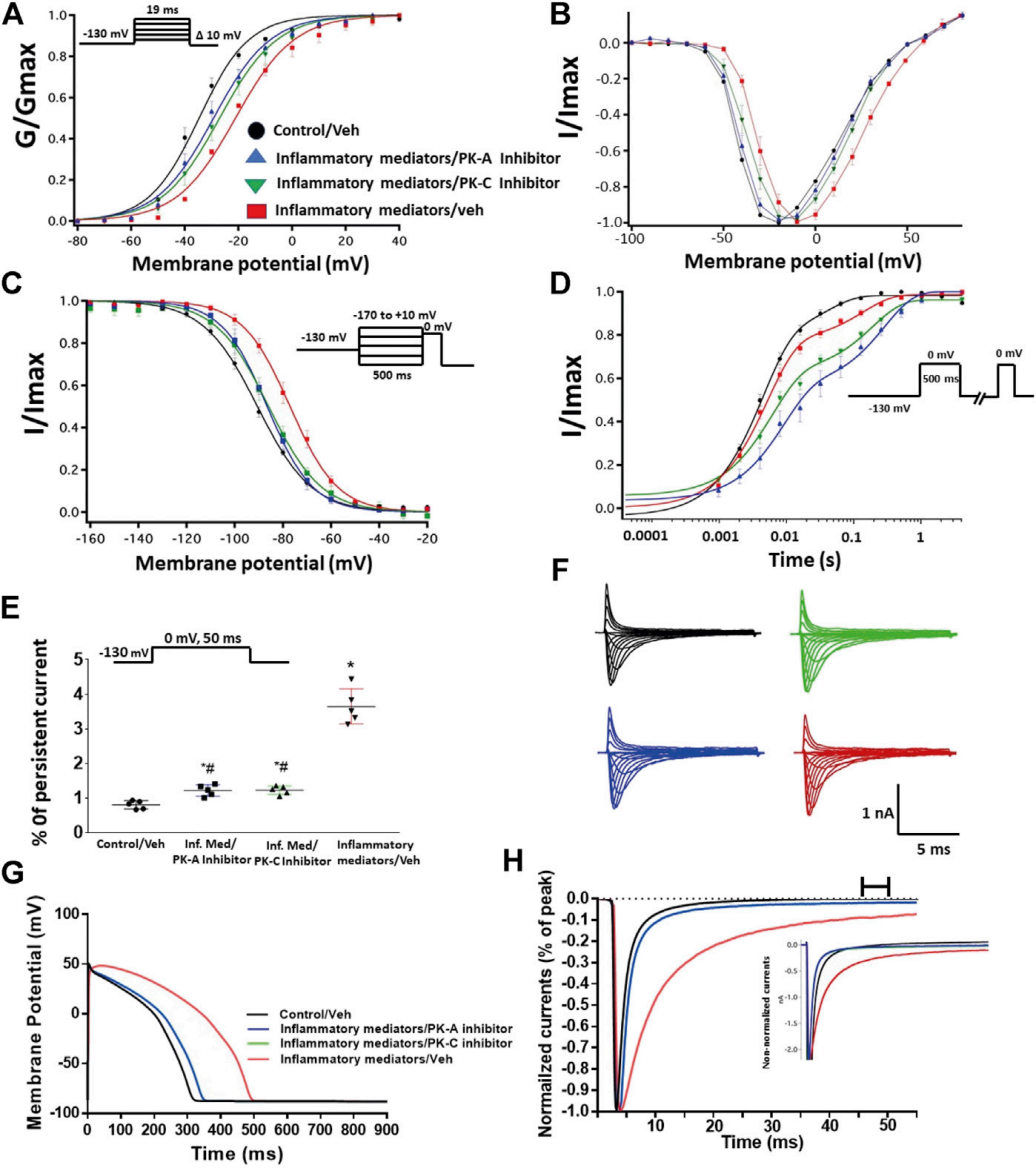
**(A)** Effect of PK-A inhibitor (H-89, 2 µM for 20 min) or PK-C inhibitor (Gö 6,983, 1 µM for 20 min) or their vehicle on the conductance curve Nav1.5 transfected CHO cells incubated in the inflammatory mediators for 24 h with the insert showing the protocol (*n* = 5, each). **(B)** IV curves **(C)** Effect of PK-A inhibitor (H-89, 2 µM for 20 min) or PK-C inhibitor (Gö 6,983, 1 µM for 20 min) or their vehicle on SSFI of Nav1.5 transfected CHO cells incubated in the inflammatory mediators for 24 h with the insert showing the protocol (*n* = 5, each). **(D)** Effect of PK-A inhibitor (H-89, 2 µM for 20 min) or PK-C inhibitor (Gö 6,983, 1 µM for 20 min) or their vehicle on recovery from fast inactivation of Nav1.5 transfected CHO cells incubated in the inflammatory mediators for 24 h with the insert showing the protocol (*n* = 5, each). **(E)** Effect of PK-A inhibitor (H-89, 2 µM for 20 min) or PK-C inhibitor (Gö 6,983, 1 µM for 20 min) or their vehicle on the percentage of persistent sodium currents of Nav1.5 transfected CHO cells incubated in the inflammatory mediators for 24 h with the insert showing the protocol (*n* = 5, each). **(F)** Representative families of macroscopic currents. **(G)** Effect of PK-A inhibitor (H-89, 2 µM for 20 min) or PK-C inhibitor (Gö 6,983, 1 µM for 20 min) on the *In silico* action potential duration of Nav1.5 transfected CHO cells incubated in inflammatory mediators for 24 h. **(H)** Representative persistent currents across conditions. Currents were normalized to peak current amplitude. Bar above current traces indicates period during which persistent current was measured Inset shows non-normalized currents. *p < 0.05 vs. corresponding “Control/Veh” values using one-way ANOVA along with post hoc testing. ^#^ *p* < 0.05 vs. corresponding “inflammatory mediators/Veh” values using Student’s t-test.

To ensure that the effects of the inflammatory mediators on Nav1.5 are indeed mediated, at least partly, through activation of PK-A and/or PK-C, we examined the effect of perfusing PK-A inhibitor [H-89, 2 µM for 20 min (Wang et al., 2013)] or PK-C inhibitor (Gö 6983, 1 µM for 20 min (Wang et al., 2013)) on Nav1.5 that had been incubated for 24 h in either inflammatory mediators or vehicle. Although H-89 or Gö 6983 had no significant effects on Nav1.5 gating under control conditions (Supplementary Figure S1; Tables 1–4), H-89 or Gö 6983 reduced the inflammatory mediator-induced shifts in V_1/2_ (H-89: *p* = 0.0108; Gö 6983: *p* = 0.0203) (Figure 3A; Table 1). Figure 3B shows IV curves. In addition, H-89 or Gö 6983 rescued the inflammatory mediator-induced shift in Nav1.5 SSFI (Figure 3C; Table 2). Moreover, H-89 or Gö 6983 (*p* = 0.0041, or *p* = 0.0017, respectively) further increased the time constant of the slow component of recovery from fast inactivation when compared to inflammatory mediators (Figure 3D; Table 3).

Figure 3E shows that H-89 or Gö 6983 (*p* < 0.0001) incompletely reduced the inflammatory mediator-induced increase in the persistent currents (Table 4). Representative families of macroscopic and persistent currents across conditions are shown (Figures 3F,H ). Importantly, *in silico* APD using the data from inhibitors of PK-A (H-89) or PK-C (Gö 6983) reduced the inflammatory mediators-induced simulated APD prolongation (Figure 3G). H-89 or Gö 6983 had no significant effect on the current density of Nav1.5 when compared to the inflammatory or control conditions (Table 1).

### Cannabidiol Rescues the Nav1.5 Gating Changes of Inflammatory Mediators, Activation of Protein Kinase A or Protein Kinase C

**FIGURE 4:**
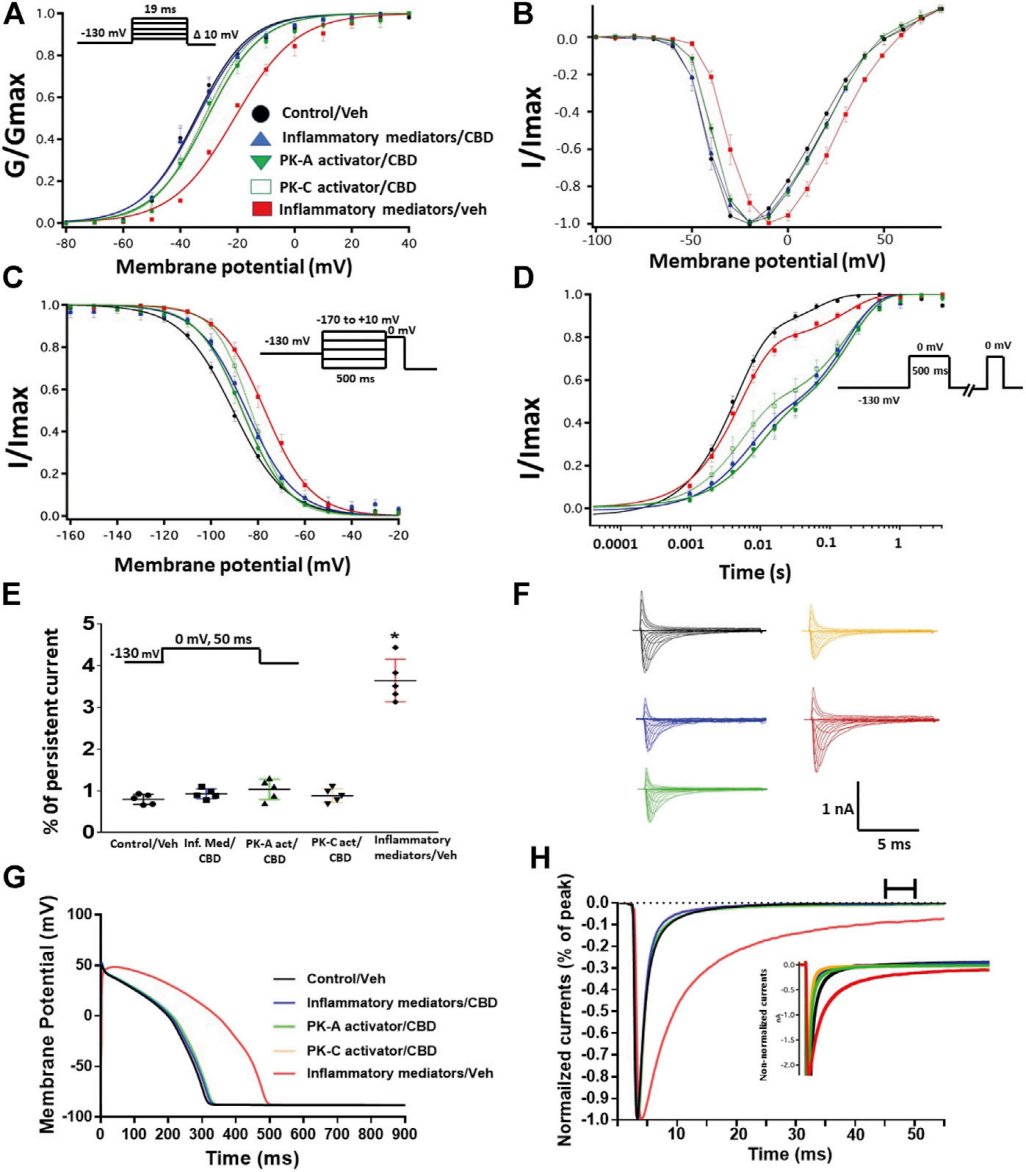
**(A)** Effect of CBD (5 μM, perfusion) on the conductance curve of Nav1.5 transfected CHO cells incubated with inflammatory mediators (24 h) or PK-A activator (CPT-cAMP; 1 μM, for 20 min) or PK-C activator (PMA; 10 nM, for 20 min) with the insert showing the protocol (*n* = 5, each). **(B)** IV curves **(C)** Effect of CBD (5 μM, perfusion) on SSFI of Nav1.5 transfected CHO cells incubated with inflammatory mediators (24 h) or PK-A activator (CPT-cAMP; 1 μM, for 20 min) or PK-C activator (PMA; 10 nM, for 20 min) with the insert showing the protocol (*n* = 5, each). **(D)** Effect of CBD (5 μM, perfusion) on recovery from fast inactivation of Nav1.5 transfected CHO cells incubated with inflammatory mediators (24 h) or PK-A activator (CPT-cAMP; 1 μM, for 20 min) or PK-C activator (PMA; 10 nM, for 20 min) with the insert showing the protocol (*n* = 5, each). **(E)** Effect of CBD (5 μM, perfusion) on the percentage of persistent sodium currents of Nav1.5 transfected CHO cells incubated with inflammatory mediators (24 h) or PK-A activator (CPT-cAMP; 1 μM, for 20 min) or PK-C activator (PMA; 10 nM, for 20 min) with the insert showing the protocol (n = 5, each). **(F)** Representative families of macroscopic currents. **(G)** Effect of CBD (5 μM, perfusion) on the *In silico* action potential duration of Nav1.5 transfected CHO cells incubated in inflammatory mediators (24 h) or PK-A activator (CPT-cAMP; 1 μM, for 20 min) or PK-C activator (PMA; 10 nM, for 20 min). **(H)** Representative persistent currents across conditions. Currents were normalized to peak current amplitude. Bar above current traces indicates period during which persistent current was measured. Inset shows non-normalized currents. *p < 0.05 vs. corresponding “Control/Veh” values using one-way ANOVA along with post hoc testing.

Coupled with our previous observation that CBD rescues high glucose-induced dysfunction in Nav1.5 (Fouda et al., 2020a), our results from the above experiments with PK-A or PK-C modulators prompted us to test the effects of CBD on the biophysical properties of Nav1.5 in the presence of inflammatory mediators, PK-C activator (PMA), or PK-A activator (CPT-cAMP). To determine whether the observed changes to activation and SSFI imparted by inflammatory mediators or activation of PK-A or PK-C could be rescued, we measured peak sodium currents in the presence of CBD. CBD concentration was selected based on its IC_50_ (Ghovanloo et al., 2018). CBD (5 µM) perfusion abolished the effects of inflammatory mediators, PMA, or CPT-cAMP, including shifts of V_1/2_ of activation, z of activation, and the V_1/2_ of SSFI (Figures 4A–C; Tables 1 Tables 2). In addition, CBD significantly increased the time constant of the slow component of recovery from fast inactivation regardless of the concurrent treatment (inflammatory mediators, PMA or CPT-cAMP) (Figure 4D; Table 3). Also, CBD significantly (*p* < 0.0001) reduced the increase in INap caused by inflammatory mediators (Figure 4E; Table 4). Also, CBD significantly (*p* < 0.0001) reduced PMA or CPT-cAMP-induced increase in INap (Figure 4E; Table 4, with representative macroscopic and persistent currents shown in Figures 4F,H). The O’Hara-Rudy model results also suggest that CBD rescues the prolonged *in silico* APD caused by inflammatory mediators or activators of PK-A or PK-C to nearly that of the control condition (Figure 4G). The reduction in APD is consistent with the anti-excitatory effects of CBD (Ghovanloo et al., 2018). CBD significantly (*p* < 0.05) reduced the current density of Nav1.5 when compared to the inflammatory or control conditions (Table 1).

**FIGURE 5:**
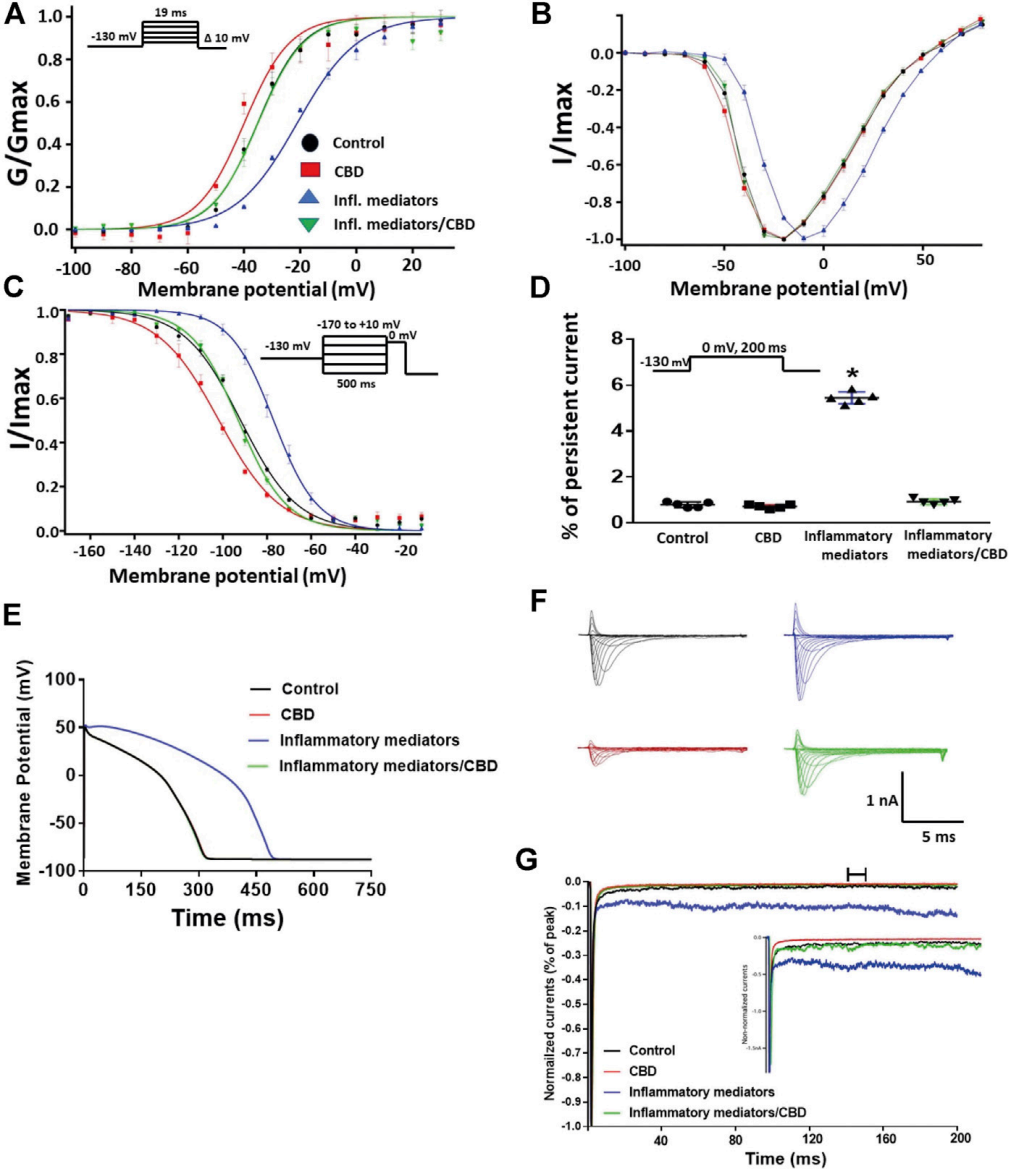
**(A)** Effect of CBD (5 μM, perfusion) or its vehicle on the conductance curve measured from human iCell cardiomyocytes (hIPSC-CMs) incubated with inflammatory mediators (24 h) or their vehicle with the insert showing the protocol (*n* = 5, each). **(B)** IV curves **(C)** Effect of CBD (5 μM, perfusion) or its vehicle on SSFI of human iCell cardiomyocytes (hIPSC-CMs) incubated with inflammatory mediators (24 h) or their vehicle with the insert showing the protocol (n = 5, each). **(D)** Effect of CBD (5 μM, perfusion) or its vehicle on the percentage of persistent sodium currents of human iCell cardiomyocytes (hIPSC-CMs) incubated with inflammatory mediators (24 h) or their vehicle with the insert showing the protocol (*n* = 5, each). **(E)** Effect of CBD (5 μM, perfusion) or its vehicle on the *In silico* action potential duration of human iCell cardiomyocytes (hIPSC-CMs) incubated in inflammatory mediators (24 h) or their vehicle. **(F)** Representative families of macroscopic currents. **(G)** Representative persistent currents across conditions. Currents were normalized to peak current amplitude. Bar above current traces indicates period during which persistent current was measured. Inset shows non-normalized currents. *p < 0.05 vs. corresponding “Control” values using one-way ANOVA along with post hoc testing.

To validate the protective effects of CBD against inflammatory mediator-induced changes observed in the CHO cells, we used hIPSC-CMs to confirm these effects. Figures 5A,B show conductance plotted as a function of membrane potential and the IV curves, respectively. We found that incubation in inflammatory mediators for 24 h significantly right-shifted V_1/2_ of activation (*p* = 0.0015) (from −37.3 ± 1.2 to −22.3 ± 2.4 mV, *n* = 5, each) and decreased z of activation curve (*p* = 0.0034) (from 3.8 ± 0.16 to 2.7 ± 0.17 mV, *n* = 5, each). Figure 5C shows normalized current amplitudes plotted as a function of pre-pulse potential. Inflammatory mediators caused significant shifts in the positive direction in the V_1/2_ obtained from Boltzmann fits (*p* = 0.0084) (from −92.3 ± 3.4 to −77.1 ± 1.7 mV, n = 5, each). To determine the effects of inflammatory mediators on the stability of Nav1.5 inactivation, we held channels at −130 mV, followed by a depolarizing pulse to 0 mV for 200 ms. Figure 5D shows that incubation in inflammatory mediators significantly increased INap compared to control (inflammatory mediators: *p* < 0.0001) (from 0.80 ± 0.05 to 5.44 ± 0.11, *n* = 5, each). While CBD had no significant effect on INap under control condition, it significantly (*p* = 0.0144) reduced the inflammatory mediators-induced increase in INap (Figure 5D). Figure 5E shows that modifying the model with data obtained from incubation in inflammatory mediators prolonged the simulated AP duration (APD) from ∼300 ms to ∼500 ms. This increased APD potentially leads to the prolongation of the QT interval (Nachimuthu et al., 2012). Representative families of macroscopic and persistent currents across conditions are shown (Figures 5F,G).

### 17β-Estradiol Rescues the High Glucose-Induced Alterations in Nav1.5 Gating via Protein Kinase A and Protein Kinase C Pathway

**FIGURE 6:**
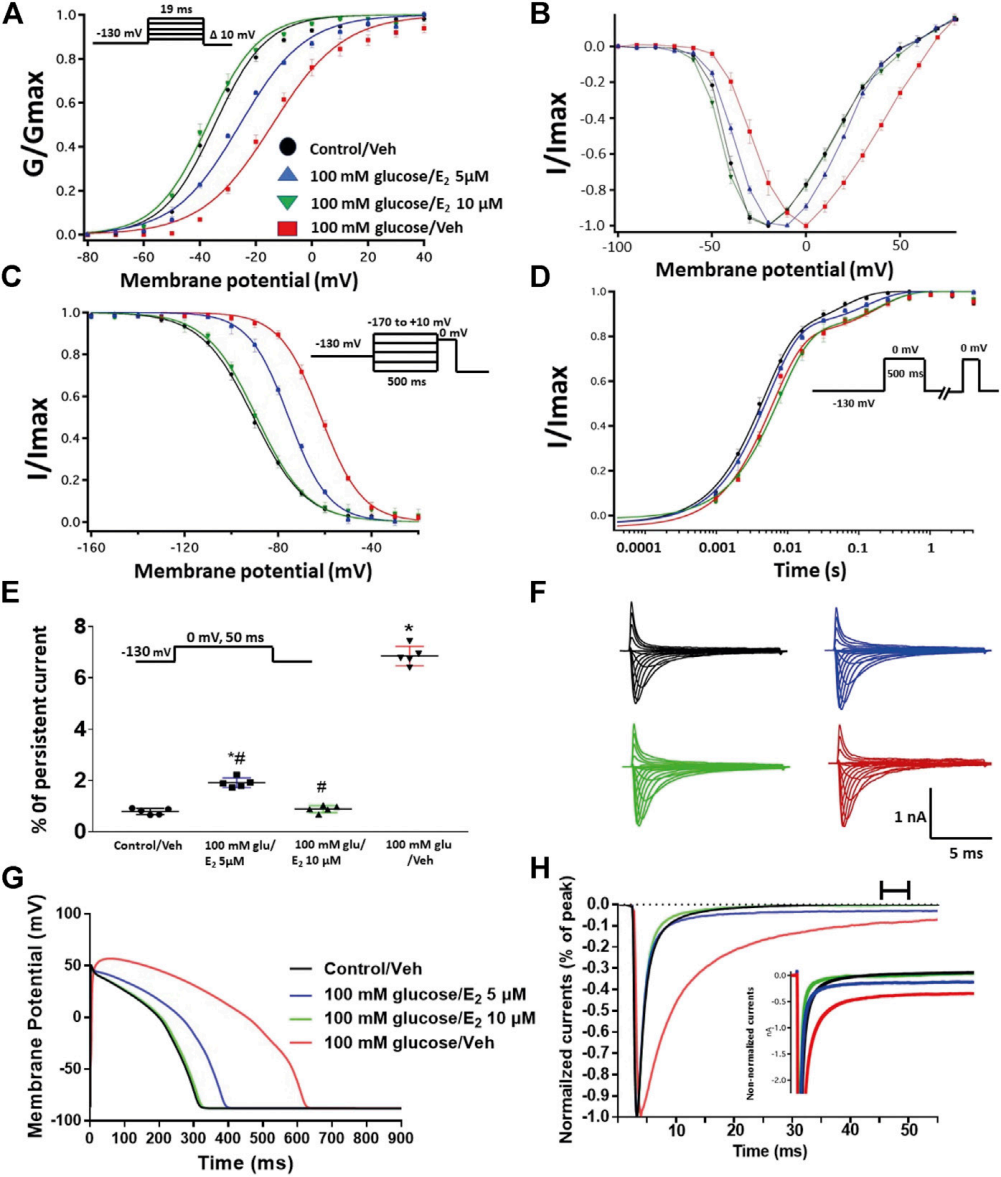
**(A)** Effect of E_2_ (5 or 10 µM) on conductance curve of Nav1.5 transfected CHO cells incubated in 100 mM glucose (for 24 h) with the insert showing the protocol (*n* = 5, each). **(B)** IV curves **(C)** Effect of E_2_ (5 or 10 µM) on SSFI of Nav1.5 transfected CHO cells in 100 mM glucose (for 24 h) with the insert showing the protocol (*n* = 5, each). **(D)** Effect of E_2_ (5 or 10 µM) on recovery from fast inactivation of Nav1.5 transfected CHO cells in 100 mM glucose (for 24 h) with the insert showing the protocol (*n* = 5, each). **(E)** Effect of E_2_ (5 or 10 µM) on the percentage of persistent sodium currents of Nav1.5 transfected CHO cells in 100 mM glucose (for 24 h) with the insert showing the protocol (*n* = 5, each). **(F)** Representative families of macroscopic currents. **(G)** Effect of E_2_ (5 or 10 µM) on the *In silico* action potential duration of Nav1.5 transfected CHO cells incubated in 100 mM glucose (for 24 h). **(H)** Representative persistent currents across conditions. Currents were normalized to peak current amplitude. Bar above current traces indicates period during which persistent current was measured. Inset shows non-normalized currents. *p < 0.05 vs. corresponding “Control/Veh” values using one-way ANOVA along with post hoc testing. ^#^ *p* < 0.05 vs. corresponding “100 mM glucose/Veh” values using Student’s t-test.

We further investigated whether E_2_ rescues the high-glucose induced changes in biophysical properties of Nav1.5 given that E_2_ previously was shown to affect Nav in addition its anti-inflammatory role (Wang et al., 2010; Iorga et al., 2017). We first tested the effects of E_2_ (5 or 10 µM) under control conditions and found that E_2_ exerted no significant effects on Nav1.5 gating (Supplementary Figure S1; Tables 1–4). In contrast, Figure 6 shows that perfusing E_2_ (5 or 10 µM) for at least 10 min into the bath solution (Möller and Netzer, 2006; Wang et al., 2013) abolished the shifts elicited by high glucose (100 mM, for 24 h, including V_1/2_, z of activation, and the V_1/2_ of SSFI in a concentration-dependent manner (Figures 6A–C; Tables 1, 2). On the other hand, we found that E_2_ (5 or10 µM) had no significant effect on 100 mM glucose-induced slight increase in the slow component of fast inactivation recovery (Figure 6D; Table 3). However, E_2_ significantly reduced the 100 mM glucose-induced increase in INap in a concentration-dependent manner (Figure 6E; Table 4). E_2_ reduction of the glucose-exacerbated INap is consistent with previous reports of similar effects in neuronal sodium channels (Wang et al., 2010). Figure 6G shows AP modeling and suggests that E_2_, in a concentration-dependent manner, rescues the prolonged *in silico* APD caused by 100 mM glucose. Representative families of macroscopic and persistent currents across conditions are shown (Figures 6F,G). E_2_ (5 or10 µM) had no significant effect on the current density of Nav1.5 when compared to the control condition or 100 mM glucose (Table 1).

**FIGURE 7:**
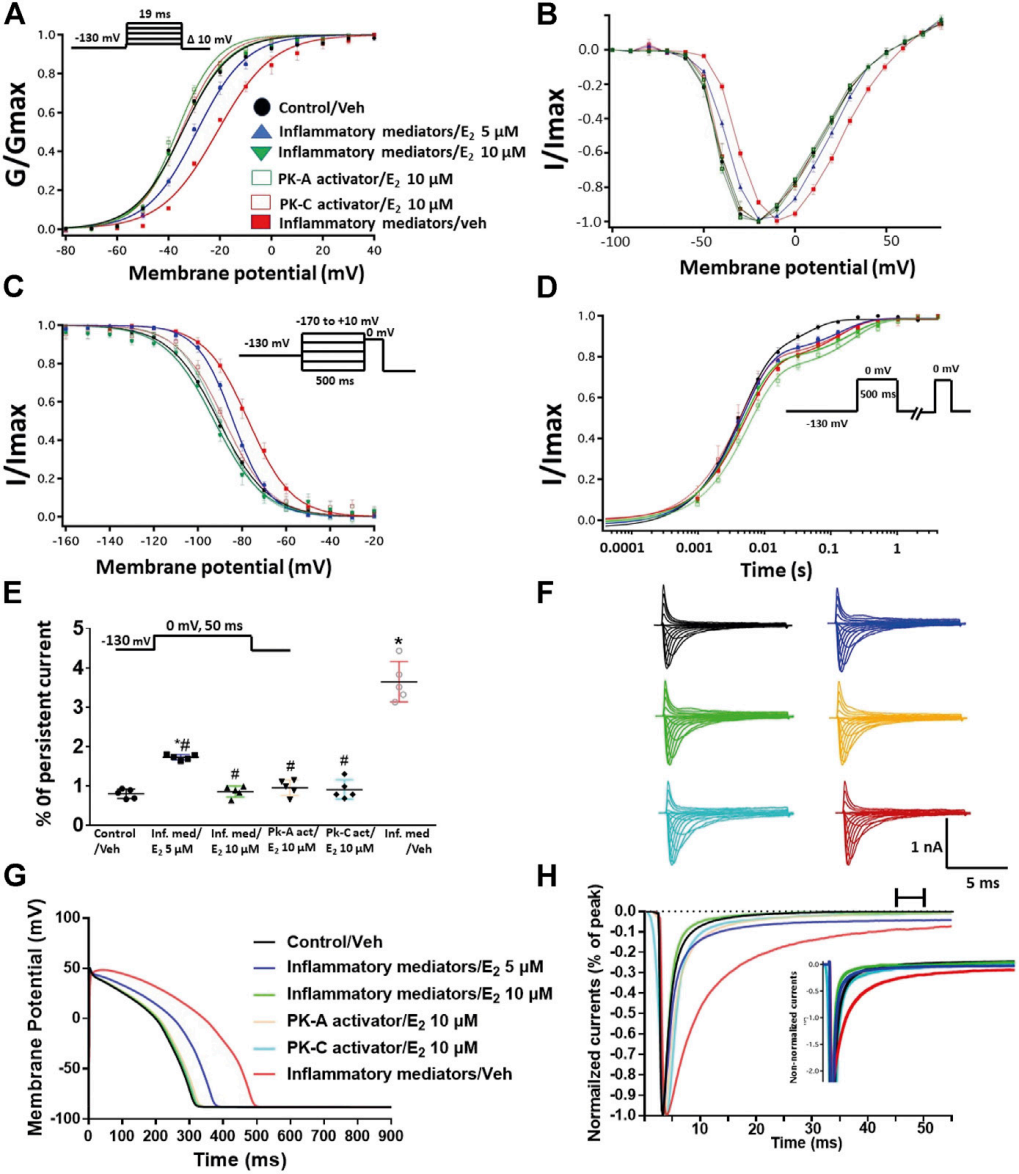
**(A)** Effect of E_2_ (5 or 10 µM) on conductance curve of Nav1.5 transfected CHO cells incubated in inflammatory mediators (for 24 h), PK-A activator (CPT-cAMP; 1 µM for 20 min) or PK-C activator (PMA; 10 nM, for 20 min) with the insert showing the protocol (n = 5, each). **(B)** IV curves **(C)** Effect of E_2_ (5 or 10 µM) on SSFI of Nav1.5 transfected CHO cells incubated in inflammatory mediators (for 24 h), PK-A activator (CPT-cAMP; 1 µM for 20 min) or PK-C activator (PMA; 10 nM, for 20 min) with the insert showing the protocol (*n* = 5, each). **(D)** Effect of E_2_ (5 or 10 µM) on recovery from fast inactivation of Nav1.5 CHO transfected cells incubated in inflammatory mediators (for 24 h), PK-A activator (CPT-cAMP; 1 µM for 20 min) or PK-C activator (PMA; 10 nM, for 20 min) with the insert showing the protocol (*n* = 5, each). **(E)** Effect of E_2_ (5 or 10 µM) on the percentage of persistent sodium currents of Nav1.5 transfected CHO cells incubated in inflammatory mediators (for 24 h), PK-A activator (CPT-cAMP; 1 µM for 20 min) or PK-C activator (PMA; 10 nM, for 20 min) with the insert showing the protocol (n = 5, each). **(F)** Representative families of macroscopic currents. **(G)** Effect of E_2_ (5 or 10 µM) on the *In silico* action potential duration of Nav1.5 transfected cells incubated in inflammatory mediators (for 24 h), PK-A activator (CPT-cAMP; 1 µM for 20 min) or PK-C activator (PMA; 10 nM, for 20 min). **(H)** Representative persistent currents across conditions. Currents were normalized to peak current amplitude. Bar above current traces indicates period during which persistent current was measured. Inset shows non-normalized currents. *p < 0.05 vs. corresponding “Control/Veh” values using one-way ANOVA along with post hoc testing. ^#^ *p* < 0.05 vs. corresponding “inflammatory mediators/Veh” values using Student’s t-test.

We tested whether E_2_ (5 or 10 µM) rescues the effects of inflammatory mediators, PK-C activator (PMA), or PK-A activator (CPT-cAMP) on the gating properties of Nav1.5. Figure 7 shows that concurrent addition of E_2_ abolished the effects of inflammatory mediators on activation and SSFI in a concentration-dependent manner (Figures 7A–C; Tables 1, 2). Similiarly, E_2_ concentration-dependently rescued PMA or CPT-cAMP-elicited effects on activation and SSFI (Figures 7A–C; Table 1, 2). Although E_2_ (5 or10 µM) had no significant effect on the slight increase in the slow component of fast inactivation recovery caused by inflammatory mediators, PMA, or CPT-cAMP (Figure 7D; Table 3), E_2_ significantly reduced the increase in INap in a concentration-dependent manner (Figure 7E; Table 4; representative currents shown in Figures 7F,H). In addition, E_2_ concentration-dependently rescues the prolonged *in silico* APD caused by inflammatory mediators or activators of PK-A or PK-C-induced to nearly that of the control condition (Figure 7G). E_2_ (5 or 10 µM) had no significant effect on the current density of Nav1.5 when compared to the control condition or the inflammatory mediaitors (Table 1).

## Discussion

**FIGURE 8:**
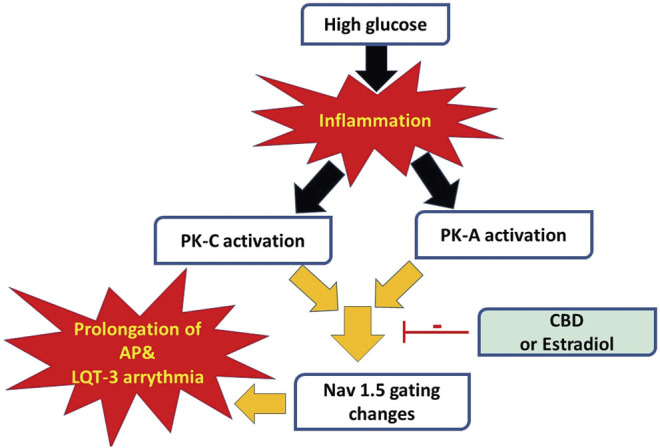
A schematic of possible cellular pathway involved in the protective effect of CBD, E_2_ against high glucose induced inflammation and activation of PK-A and PK-C via affecting cardiac voltage-gated sodium channels (Nav1.5).

We recently showed that CBD confers protection on Nav1.5 against the high glucose-elicited hyperexictability and cytotoxicity (Fouda et al., 2020a). Here, we address, for the first time, the inflammation/PK-A and PK-C signaling pathway to mediate high glucose-induced cardiac anomalies (Figure 8). Our results suggest that CBD and E_2_ may exert their cardioprotective effects against high glucose, at least partly, through this signaling pathway. Our conclusions are based on the following observations: 1) Similar to high glucose, inflammatory mediators elicited right shifts in the voltage-dependence of activation and inactivation, and exacerbated persistent currents. Increased persistent currents prolong the simulated action potential duration. 2) Activators of PK-A and PK-C reproduced the high glucose- and inflammation-induced changes in Nav1.5 gating. 3) Inhibitors of PK-A and PK-C reduced, to a great extent, the high glucose- and inflammation-induced changes in Nav1.5 gating. 4) CBD or E_2_ rescued the effects of high glucose, inflammatory mediators, or PK-A or PK-C activators. Our results suggest a role for Nav1.5 in high glucose induced hyperexcitability, via inflammation and subsequent activation of PK-A and PK-C, which could lead to LQT3-type arrhythmia (Figure 8). In addition, our findings suggest possible therapeutic effects for CBD in high glucose-provoked cardiac dysfunction in diabetic patients, especially those post-menopause.

Diabetes-induced QT prolongation predisposes to malignant ventricular arrhythmias (Ukpabi and Onwubere, 2017). Moreover, LQT in diabetic patients make them three times more vulnerable to the risk of cardiac arrest (Whitsel et al., 2005). Nav1.5 gain-of-function plays a crucial role in the development of LQT (Shimizu and Antzelevitch, 1999). With that in mind, we found that inflammatory mediators replicated the high glucose-induced changes in Nav1.5 gating similar to those correlated with LQT3 in diabetic rats (Yu et al., 2018). This finding is consistent with other reports showing that hyperglycemia/high glucose is proinflammatory and that inflammation is a crucial player in the pathogenesis of cardiovascular anamolies (Tsalamandris et al., 2019; Fouda et al., 2020b). Accumulating evidence shows that inflammation is a potential cause for developing LQT through direct effects on myocardial electric properties, including its effect on Nav, and indirect autonomic cardiac regulations (Lazzerini et al., 2015). Inflammation alters the electrophysiological properties of iCell cardiomyocytes Nav with an increase in INap leading to prolongation of APD, similar to our findings (Figure 1) (Ward et al., 2006; Shryock et al., 2013). Taken together, these findings support our hypothesis that high glucose, at least partly through induction of inflammation, alters Nav1.5 gating and leads to LQT arrhythmia (Figure 8).

The activation of PK-A and PK-C and subsequent protein phosphorylation are among the key signaling pathways associated with inflammation (Karin, 2005) and hyperglycemia, resulting in many devastating diabetes-induced cardiac complications (Koya and King, 1998; Bockus and Humphries, 2015). Our data suggest that activation of PK-A or PK-C replicated high glucose- and inflammation-induced gating changes in Nav1.5 gating, whereas inhibition of PK-A or PK-C abolished those changes (Figures 2, 3). This finding suggests that PK-A and PK-C may be downstream effectors of inflammation in high glucose-induced cardiac complications (Figure 8). PK-A phosphorlylates S525 and S528, while PK-C phosphorylates S1503 in human Nav1.5 (Iqbal and Lemmens-Gruber, 2019). There are conflicting reports regarding the effects of PK-A and PK-C activation on the voltage-dependence and kinetics of Nav1.5 gating. These differences could be attributed to different voltage protocols, different holding potentials, different concentrations or type of PK-activators, or different cell lines used in the various studies (Aromolaran et al., 2018; Iqbal and Lemmens-Gruber, 2019). Despite these discrepancies, both PK-A or PK-C destabilize Nav fast inactivation and hence increase INap, which is strongly correlated to prolonged APD as shown in our findings (Figure 8) (Astman et al., 1998; Franceschetti et al., 2000; Tateyama et al., 2003).

Our results with PK-A and PK-C modulators prompted us to test whether CBD affects the biophysical properties of Nav1.5 through this pathway. Notably, CBD has little to no affinity for endocannabinoid receptors (Thomas et al., 1998). We investigated the possible protective effect of CBD against the deletrious effects of high glucose through this signaling pathway because CBD protects against high glucose-induced gating changes in Nav1.5 (Fouda et al., 2020a). In addition, CBD attenuates the diabetes-induced inflammation and subsequent cardiac fibrosis through inhibition of phosphorylation enzymes (such as MAPKs) (Rajesh et al., 2010). Our results suggest that CBD alleviates the inflammation/activation of PK-A or PK-C induced biophyscial changes (Figure 4). Our findings are consistent with the anti-inflammatory, anti-oxidant, and anti-tumor effects of CBD via inhibition of PK-A and PK-C signaling (Seltzer et al., 2020). The incomplete protective effects of PK-A and PK-C inhibitors compared to the CBD effect against the inflammation-induced gating changes in Nav1.5 could be attributed to the direct inhibitory effect CBD has on Nav1.5 (Ghovanloo et al., 2018; Fouda et al., 2020a), among other possible contributing pathways, including phosphorylation. These potential mechanisms need further investigation.

Interestingly, E_2_ directly inhibits Nav and exerts anti-inflammatory effects (Wang et al., 2010; Iorga et al., 2017). We found that E_2_, similar to CBD, rescues the effects of high-glucose, inflammation, and activation of PK-A or PK-C (Figures 6–8). Our results are consistent with other reports showing the cardioprotective effects of E_2_ by increasing angiogenesis, vasodilation, and decreasing oxidative stress and fibrosis (Iorga et al., 2017). Although the role of E_2_ in arrhythmias is controversial, many studies support the anti-arrythmic effects of E_2_ because of its effects on the expression and function of cardiac ion channels (Odening and Koren, 2014; Iorga et al., 2017). Notably, E_2_ stabilizes Nav fast inactivation and reduces INap, similar to CBD (Wang et al., 2010). Further, E_2_ reduces the oxidative stress and the inflammatory reponses by inhibiting PK-A and PK-C-mediated signaling pathways (Viviani et al., 2002; Mize et al., 2003). Notably, CHO cells endogenously express E_2_ receptors alpha and beta (Thomas et al., 2003). It is important to note that E_2_ activates intracellular signals by two pathways: genomic (Hall and Korach, 2002) or nongenomic activation (Watson et al., 2007). E_2_ binds to the intracellular E_2_ receptors (ERs) in the cytosol or nucleus controling the gene expression in the genomic pathway while it activates fast intracellular signals through the non-genomic pathway (Zhang et al., 2012). E_2_ affects the ion channel function via a non-genomic mechanism such as transient receptor potential vanilloid receptor 1 (Xu et al., 2008), calcium channels (Lee et al., 2002) and Nav (Wang et al., 2013). In addition, our findings are consistent with others showing that E_2_ affects Nav in a concentration-dependent, rapid, reversible manner and dependent on a PKC–PKA signaling pathway (Wang et al., 2013).

Our results suggest that inflammation and the subsequent activation of PK-A and PK-C correlates with the high glucose-induced electrophysiological changes in Nav1.5 gating (Figure 8). Future studies may determine whether there is a causal relationship between elevated glucose and PK-A and/or PK-C mediated channel phosphorylation. *In silico*, these gating changes result in prolongation of simulated action potentials leading to LQT3 arrhythmia, which is a clinical complication of diabetes (Grisanti, 2018). A caveat of the AP modeling is that only sodium channel properties were modified. The properties of other channels were left unchanged even though previous reports show they are affected by the experimental parameters (Ali et al., 2015; Le Marois et al., 2020; Orvos et al., 2020).

In conclusion, CBD and E_2_, through inhibition of the PK-A and PK-C signaling pathways, appear to ameliorate the effects of high glucose and the resultant clinical condition. In light of the debate about the risks associated with hormonal replacement therapy (Climént-Palmer and Spiegelhalter, 2019), CBD may provide an alternate therapeutic approach, especially in diabetic post-menopausal populations due to their decreased levels of cardioprotective E_2_ (Xu et al., 2014).

## Data Availability

The original contributions presented in the study are included in the article/Supplementary Material, further inquiries can be directed to the corresponding author.

## Abbreviations

Nav, voltage-gated sodium channel; CHO, chinese hamster ovary cells; CBD, cannabidiol; LQT3, long QT syndrome type 3; PK-A, protein kinase A; PK-C, protein kinase C; E2, 17β-estradiol; G, conductance; SSFI, steady-state fast inactivation; INap, late (or persistent) sodium current; APD, action potential duration; AP, action potential.
