# Supplementary figures and images for: Protein Kinases Mediate Anti-Inflammatory Effects of Cannabidiol and Estradiol Against High Glucose in Cardiac Sodium Channels
Source: Front Pharmacol. 2021 Apr 28;12:668657. doi: 10.3389/fphar.2021.668657 (PMC8115126; doi:10.3389/fphar.2021.668657)

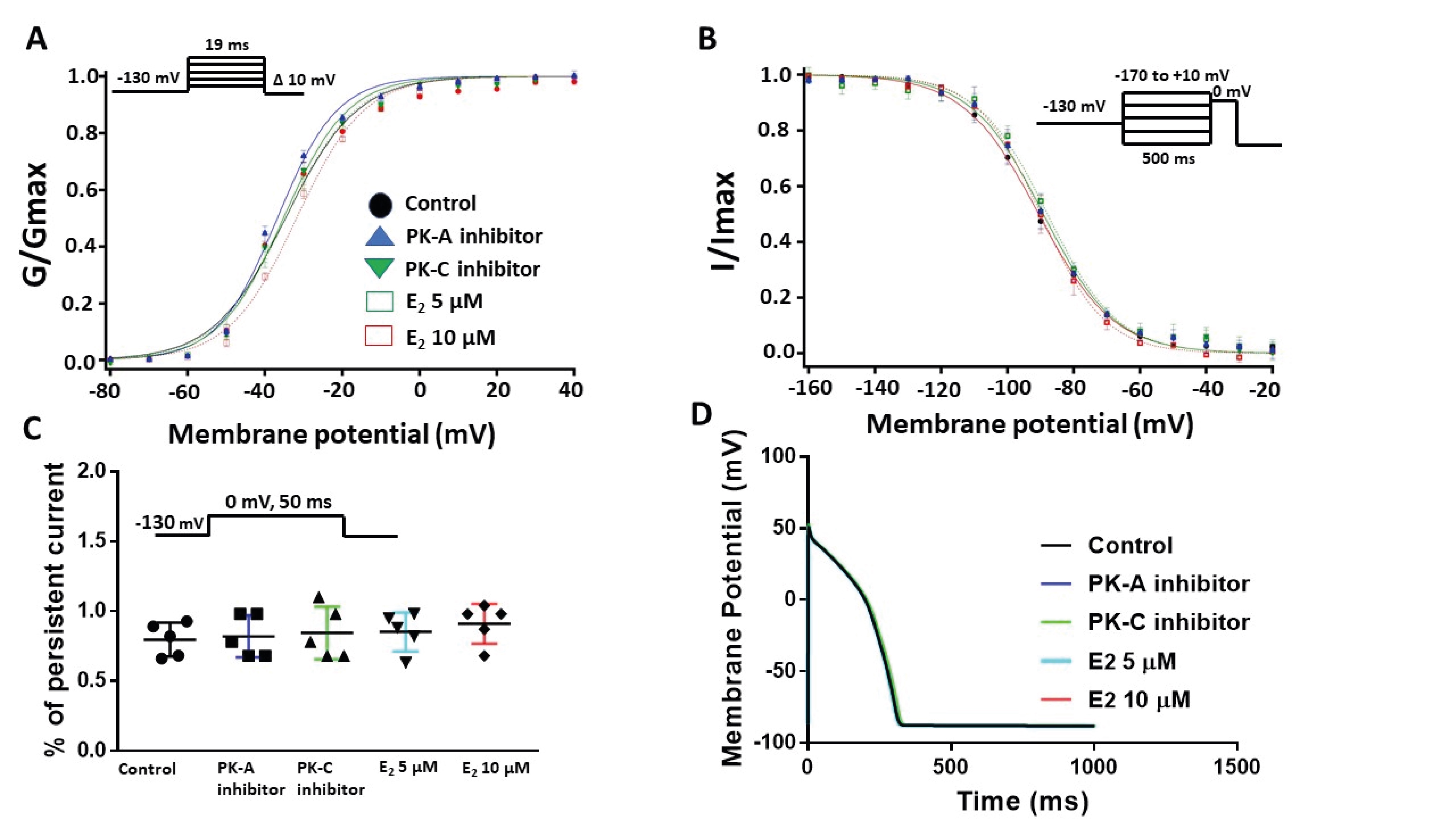

Supplement: Supplementary file 1 [file Image1.JPEG]
